# {*N*′-[(*E*)-(5-Bromo-2-oxidophen­yl)(phen­yl)methyl­ene]benzohydrazidato}pyridine­nickel(II)

**DOI:** 10.1107/S1600536809028207

**Published:** 2009-07-22

**Authors:** Chang-Zheng Zheng, Chang-You Ji, Xiu-Li Chang, Chao-Hua Zhang

**Affiliations:** a College of Environmental and Chemical Engineering, Xi’an Polytechnic University, Xi’an, Shaanxi 710048, People’s Republic of China; b School of Chemistry & Pharmaceutical Engineering, Sichuan University of Science & Engineering, Zigong, Sichuan 643000, People’s Republic of China; c Liaoning Key Laboratory of Applied Chemistry, Institute of Superfine Chemicals, Bohai University, Jinzhou 121000, People’s Republic of China

## Abstract

The asymmetric unit of title complex, [Ni(C_20_H_13_BrN_2_O_2_)(C_5_H_5_N)], contains two independent mol­ecules. In each mol­ecule, the central Ni^II^ atom has a square-planar environment, formed by the tridentate hydrazone and the monodentate pyridine ligands, with the N atoms in a *trans* arrangement about the Ni^II^ atom.

## Related literature

For the coordination properties of aroylhydrazones, see: Ali *et al.* (2004 ▶); Carcelli *et al.* (1995 ▶); Salem (1998 ▶); Singh *et al.* (1982 ▶).
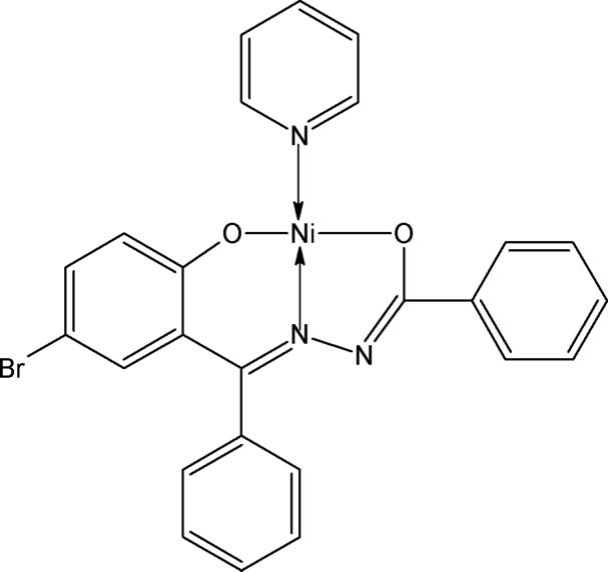


## Experimental

### 

#### Crystal data

[Ni(C_20_H_13_BrN_2_O_2_)(C_5_H_5_N)]
*M* *_r_* = 531.01
Monoclinic, 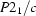

*a* = 22.638 (5) Å
*b* = 10.628 (2) Å
*c* = 19.302 (4) Å
β = 108.597 (4)°
*V* = 4401.5 (16) Å^3^
*Z* = 8
Mo *K*α radiation
μ = 2.73 mm^−1^
*T* = 295 K
0.16 × 0.12 × 0.08 mm

#### Data collection

Bruker APEXII CCD area-detector diffractometer
Absorption correction: multi-scan (*SADABS*; Bruker, 2005 ▶) *T*_min_ = 0.670, *T*_max_ = 0.812
22758 measured reflections
7792 independent reflections
4790 reflections with *I* > 2σ(*I*)
*R*_int_ = 0.054

#### Refinement

*R*[*F* ^2^ > 2σ(*F* ^2^)] = 0.045
*wR*(*F* ^2^) = 0.120
*S* = 1.01
7792 reflections
577 parameters
H-atom parameters constrained
Δρ_max_ = 0.98 e Å^−3^
Δρ_min_ = −0.64 e Å^−3^

### 

Data collection: *APEX2* (Bruker, 2005 ▶); cell refinement: *SAINT* (Bruker, 2005 ▶); data reduction: *SAINT*; program(s) used to solve structure: *SHELXS97* (Sheldrick, 2008 ▶); program(s) used to refine structure: *SHELXL97* (Sheldrick, 2008 ▶); molecular graphics: *SHELXTL* (Sheldrick, 2008 ▶); software used to prepare material for publication: *SHELXTL*.

## Supplementary Material

Crystal structure: contains datablocks I, global. DOI: 10.1107/S1600536809028207/rk2149sup1.cif

Structure factors: contains datablocks I. DOI: 10.1107/S1600536809028207/rk2149Isup2.hkl

Additional supplementary materials:  crystallographic information; 3D view; checkCIF report

## Figures and Tables

**Fig. 1.**
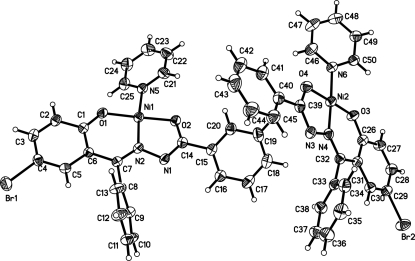
The asymmetric unit of title compound, showing the atom–numbering scheme. Displacement ellipsoids are drawn at the 30% probability level. H atoms are presented as a small spheres of arbitrary radius.

## supplementary crystallographic information

### Comment

The chemistry of aroylhydrazones continues to attract much attention due to their coordination ability to metal ions (Singh *et al.*, 1982; Salem, 1998; Ali *et al.*, 2004.) and their biological activity (Singh *et al.*, 1982; Carcelli *et al.*, 1995.) As an extension of work on the structural characterisation of aroylhydrazone derivatives, the title compound, {*N*–[(*E*)–(5-bromo–2–hydroxyphenyl)– (phenyl)methylene]benzohydrazide}pyridinenickel(II) (Fig. 1) was synthesized and its crystal structure is reported here.

### Experimental

A *DMF* solution (5 ml) of *N*–[(*E*)–(5–bromo–2–hydroxyphenyl)– (phenyl)methylene]benzohydrazide (0.25 mmol, 0.099 g) was mixed with a methanol solution(5 ml) of NiCl_2_^.^6H_2_O (0.25 mmol, 0.059 g). The mixture was stirred at 298 K for 4 h and then filtered. A red precipitate was produced after about 10 d. A pyridine mixture (5 ml) was used to dissolve the precipitate at 330 K. A red block–shaped crystals were obtained after one month (yield 30%).

### Crystal data

**Table:**

[Ni(C_20_H_13_BrN_2_O_2_)(C_5_H_5_N)]	*F*(000) = 2144
*M**_r_* = 531.01	*D*_x_ = 1.603 Mg m^−^^3^
Monoclinic, *P*2_1_/*c*	Melting point: 330 K
Hall symbol: -P 2ybc	Mo *K*α radiation, λ = 0.71073 Å
*a* = 22.638 (5) Å	Cell parameters from 3249 reflections
*b* = 10.628 (2) Å	θ = 2.7–20.5°
*c* = 19.302 (4) Å	µ = 2.73 mm^−^^1^
β = 108.597 (4)°	*T* = 295 K
*V* = 4401.5 (16) Å^3^	Block, red
*Z* = 8	0.16 × 0.12 × 0.08 mm

### Data collection

**Table:**

Bruker APEXII CCD area-detector diffractometer	7792 independent reflections
Radiation source: fine–focus sealed tube	4790 reflections with *I* > 2σ(*I*)
graphite	*R*_int_ = 0.054
φ and ω scans	θ_max_ = 25.1°, θ_min_ = 1.0°
Absorption correction: multi-scan (*SADABS*; Bruker, 2005)	*h* = −26→26
*T*_min_ = 0.670, *T*_max_ = 0.812	*k* = −11→12
22758 measured reflections	*l* = −22→22

### Refinement

**Table:**

Refinement on *F*^2^	Primary atom site location: structure-invariant direct methods
Least-squares matrix: full	Secondary atom site location: difference Fourier map
*R*[*F*^2^ > 2σ(*F*^2^)] = 0.045	Hydrogen site location: inferred from neighbouring sites
*wR*(*F*^2^) = 0.120	H-atom parameters constrained
*S* = 1.00	*w* = 1/[σ^2^(*F*_o_^2^) + (0.0499*P*)^2^ + 2.599*P*] where *P* = (*F*_o_^2^ + 2*F*_c_^2^)/3
7792 reflections	(Δ/σ)_max_ = 0.001
577 parameters	Δρ_max_ = 0.98 e Å^−^^3^
0 restraints	Δρ_min_ = −0.64 e Å^−^^3^

### Special details

**Table:**

Geometry. All s.u.'s (except the s.u. in the dihedral angle between two l.s. planes) are estimated using the full covariance matrix. The cell s.u.'s are taken into account individually in the estimation of s.u.'s in distances, angles and torsion angles; correlations between s.u.'s in cell parameters are only used when they are defined by crystal symmetry. An approximate (isotropic) treatment of cell s.u.'s is used for estimating s.u.'s involving l.s. planes.
Refinement. Refinement of *F*^2^ against ALL reflections. The weighted *R*–factor *wR* and goodness of fit *S* are based on *F*^2^, conventional *R*–factors *R* are based on *F*, with *F* set to zero for negative *F*^2^. The threshold expression of *F*^2^ > σ(*F*^2^) is used only for calculating *R*–factors(gt) *etc*. and is not relevant to the choice of reflections for refinement. *R*–factors based on *F*^2^ are statistically about twice as large as those based on *F*, and *R*–factors based on ALL data will be even larger.

### Fractional atomic coordinates and isotropic or equivalent isotropic displacement parameters (Å^2^)

**Table:**

	*x*	*y*	*z*	*U*_iso_*/*U*_eq_	
Ni1	0.45264 (3)	0.42546 (5)	0.35981 (3)	0.04172 (18)	
Ni2	0.06865 (3)	0.81105 (6)	0.09736 (3)	0.04584 (19)	
Br1	0.75838 (3)	0.56368 (6)	0.62155 (3)	0.0690 (2)	
Br2	0.08512 (3)	1.22986 (6)	−0.19492 (3)	0.0750 (2)	
O1	0.51622 (15)	0.3521 (3)	0.42990 (17)	0.0482 (9)	
O2	0.38672 (15)	0.5023 (3)	0.29245 (16)	0.0459 (8)	
O3	0.04179 (16)	0.8259 (3)	−0.00067 (17)	0.0531 (9)	
O4	0.09816 (16)	0.7926 (3)	0.19701 (17)	0.0528 (9)	
N1	0.45027 (19)	0.6751 (4)	0.3254 (2)	0.0454 (10)	
N2	0.48688 (19)	0.5832 (3)	0.3720 (2)	0.0424 (10)	
N3	0.14700 (19)	0.9788 (4)	0.1884 (2)	0.0481 (10)	
N4	0.11546 (18)	0.9555 (4)	0.1141 (2)	0.0445 (10)	
N5	0.4137 (2)	0.2629 (4)	0.3344 (2)	0.0451 (10)	
N6	0.0205 (2)	0.6599 (4)	0.0883 (2)	0.0479 (10)	
C1	0.5666 (2)	0.4064 (4)	0.4720 (2)	0.0407 (12)	
C2	0.6088 (2)	0.3323 (5)	0.5247 (3)	0.0487 (13)	
H2	0.5983	0.2490	0.5296	0.058*	
C3	0.6643 (3)	0.3755 (5)	0.5691 (3)	0.0525 (14)	
H3	0.6913	0.3226	0.6031	0.063*	
C4	0.6800 (2)	0.5010 (5)	0.5629 (3)	0.0465 (13)	
C5	0.6392 (2)	0.5776 (5)	0.5140 (3)	0.0441 (12)	
H5	0.6501	0.6613	0.5112	0.053*	
C6	0.5815 (2)	0.5355 (4)	0.4681 (2)	0.0381 (11)	
C7	0.5394 (2)	0.6220 (4)	0.4173 (2)	0.0382 (11)	
C8	0.5575 (2)	0.7570 (4)	0.4182 (2)	0.0397 (11)	
C9	0.5777 (2)	0.8056 (5)	0.3638 (3)	0.0524 (13)	
H9	0.5778	0.7555	0.3244	0.063*	
C10	0.5978 (3)	0.9289 (5)	0.3675 (3)	0.0611 (15)	
H10	0.6121	0.9609	0.3309	0.073*	
C11	0.5970 (3)	1.0040 (5)	0.4246 (3)	0.0625 (16)	
H11	0.6108	1.0868	0.4271	0.075*	
C12	0.5758 (3)	0.9569 (5)	0.4778 (3)	0.0674 (17)	
H12	0.5746	1.0081	0.5164	0.081*	
C13	0.5561 (3)	0.8340 (5)	0.4747 (3)	0.0547 (14)	
H13	0.5418	0.8027	0.5113	0.066*	
C14	0.3990 (2)	0.6205 (5)	0.2858 (3)	0.0439 (12)	
C15	0.3525 (2)	0.6938 (4)	0.2296 (2)	0.0439 (12)	
C16	0.3697 (3)	0.7823 (5)	0.1885 (3)	0.0534 (14)	
H16	0.4117	0.7997	0.1972	0.064*	
C17	0.3247 (3)	0.8469 (5)	0.1335 (3)	0.0658 (17)	
H17	0.3367	0.9064	0.1053	0.079*	
C18	0.2630 (3)	0.8227 (6)	0.1214 (3)	0.0649 (16)	
H18	0.2328	0.8650	0.0846	0.078*	
C19	0.2459 (3)	0.7356 (5)	0.1637 (3)	0.0662 (16)	
H19	0.2038	0.7204	0.1563	0.079*	
C20	0.2902 (3)	0.6707 (5)	0.2168 (3)	0.0528 (14)	
H20	0.2781	0.6104	0.2444	0.063*	
C21	0.3517 (3)	0.2521 (5)	0.3074 (3)	0.0586 (15)	
H21	0.3276	0.3246	0.3016	0.070*	
C22	0.3222 (3)	0.1399 (6)	0.2881 (3)	0.0726 (17)	
H22	0.2790	0.1363	0.2694	0.087*	
C23	0.3569 (3)	0.0329 (6)	0.2967 (3)	0.0733 (18)	
H23	0.3376	−0.0450	0.2845	0.088*	
C24	0.4199 (3)	0.0412 (5)	0.3230 (3)	0.0665 (17)	
H24	0.4444	−0.0308	0.3292	0.080*	
C25	0.4470 (3)	0.1572 (5)	0.3405 (3)	0.0575 (15)	
H25	0.4903	0.1627	0.3573	0.069*	
C26	0.0544 (2)	0.9173 (5)	−0.0393 (3)	0.0445 (12)	
C27	0.0255 (2)	0.9109 (5)	−0.1155 (3)	0.0513 (13)	
H27	−0.0002	0.8426	−0.1348	0.062*	
C28	0.0335 (2)	1.0006 (5)	−0.1624 (3)	0.0550 (14)	
H28	0.0134	0.9946	−0.2124	0.066*	
C29	0.0727 (2)	1.1011 (5)	−0.1327 (3)	0.0485 (13)	
C30	0.1021 (2)	1.1097 (5)	−0.0600 (3)	0.0458 (12)	
H30	0.1283	1.1777	−0.0421	0.055*	
C31	0.0942 (2)	1.0188 (4)	−0.0103 (2)	0.0421 (12)	
C32	0.1244 (2)	1.0355 (4)	0.0670 (3)	0.0423 (12)	
C33	0.1665 (2)	1.1473 (5)	0.0940 (3)	0.0466 (13)	
C34	0.1508 (3)	1.2401 (5)	0.1358 (3)	0.0575 (14)	
H34	0.1154	1.2313	0.1497	0.069*	
C35	0.1880 (3)	1.3452 (6)	0.1565 (3)	0.0710 (17)	
H35	0.1776	1.4078	0.1843	0.085*	
C36	0.2398 (3)	1.3567 (6)	0.1360 (4)	0.0786 (19)	
H36	0.2649	1.4275	0.1501	0.094*	
C37	0.2558 (3)	1.2651 (6)	0.0947 (3)	0.0729 (18)	
H37	0.2914	1.2742	0.0810	0.087*	
C38	0.2193 (3)	1.1616 (5)	0.0740 (3)	0.0556 (14)	
H38	0.2301	1.0998	0.0461	0.067*	
C39	0.1347 (2)	0.8865 (5)	0.2267 (3)	0.0479 (13)	
C40	0.1637 (2)	0.8869 (5)	0.3062 (3)	0.0511 (14)	
C41	0.1625 (3)	0.7808 (6)	0.3465 (3)	0.0635 (16)	
H41	0.1416	0.7094	0.3235	0.076*	
C42	0.1923 (3)	0.7798 (7)	0.4212 (3)	0.0754 (18)	
H42	0.1915	0.7074	0.4480	0.090*	
C43	0.2224 (3)	0.8831 (8)	0.4554 (3)	0.086 (2)	
H43	0.2424	0.8806	0.5056	0.103*	
C44	0.2240 (3)	0.9913 (7)	0.4179 (3)	0.084 (2)	
H44	0.2442	1.0626	0.4420	0.100*	
C45	0.1948 (3)	0.9922 (6)	0.3428 (3)	0.0655 (16)	
H45	0.1961	1.0648	0.3164	0.079*	
C46	0.0367 (3)	0.5674 (5)	0.1383 (3)	0.0603 (15)	
H46	0.0733	0.5757	0.1775	0.072*	
C47	0.0011 (3)	0.4611 (5)	0.1337 (3)	0.0710 (18)	
H47	0.0138	0.3983	0.1688	0.085*	
C48	−0.0532 (3)	0.4483 (5)	0.0768 (3)	0.0689 (17)	
H48	−0.0783	0.3776	0.0731	0.083*	
C49	−0.0696 (3)	0.5409 (5)	0.0261 (3)	0.0593 (15)	
H49	−0.1064	0.5346	−0.0130	0.071*	
C50	−0.0321 (3)	0.6433 (5)	0.0323 (3)	0.0525 (14)	
H50	−0.0435	0.7045	−0.0040	0.063*	

### Atomic displacement parameters (Å^2^)

**Table:**

	*U*^11^	*U*^22^	*U*^33^	*U*^12^	*U*^13^	*U*^23^
Ni1	0.0482 (4)	0.0334 (3)	0.0423 (4)	0.0018 (3)	0.0126 (3)	0.0002 (3)
Ni2	0.0512 (4)	0.0451 (4)	0.0404 (4)	−0.0004 (3)	0.0134 (3)	0.0040 (3)
Br1	0.0527 (4)	0.0775 (4)	0.0669 (4)	0.0002 (3)	0.0053 (3)	0.0001 (3)
Br2	0.0966 (5)	0.0765 (4)	0.0472 (3)	−0.0218 (4)	0.0162 (3)	0.0117 (3)
O1	0.054 (2)	0.0325 (19)	0.053 (2)	−0.0032 (16)	0.0097 (18)	0.0049 (16)
O2	0.052 (2)	0.035 (2)	0.045 (2)	−0.0031 (16)	0.0067 (16)	−0.0012 (16)
O3	0.067 (2)	0.048 (2)	0.0408 (19)	−0.0117 (18)	0.0115 (17)	0.0047 (17)
O4	0.056 (2)	0.058 (2)	0.043 (2)	−0.0069 (19)	0.0137 (17)	0.0020 (18)
N1	0.053 (3)	0.034 (2)	0.046 (2)	0.006 (2)	0.012 (2)	0.006 (2)
N2	0.052 (3)	0.036 (2)	0.039 (2)	0.003 (2)	0.013 (2)	0.0034 (19)
N3	0.051 (3)	0.052 (3)	0.037 (2)	0.002 (2)	0.009 (2)	0.003 (2)
N4	0.043 (3)	0.053 (3)	0.035 (2)	0.004 (2)	0.0089 (19)	0.003 (2)
N5	0.053 (3)	0.038 (2)	0.044 (2)	0.000 (2)	0.015 (2)	−0.0012 (19)
N6	0.056 (3)	0.044 (3)	0.045 (2)	0.001 (2)	0.018 (2)	0.005 (2)
C1	0.046 (3)	0.039 (3)	0.039 (3)	0.005 (2)	0.016 (2)	−0.001 (2)
C2	0.060 (4)	0.035 (3)	0.048 (3)	0.007 (3)	0.014 (3)	0.003 (2)
C3	0.057 (4)	0.053 (4)	0.045 (3)	0.012 (3)	0.013 (3)	0.004 (3)
C4	0.043 (3)	0.056 (4)	0.039 (3)	0.002 (3)	0.012 (2)	−0.004 (3)
C5	0.049 (3)	0.042 (3)	0.046 (3)	−0.003 (3)	0.020 (3)	−0.001 (2)
C6	0.040 (3)	0.040 (3)	0.035 (3)	0.000 (2)	0.013 (2)	−0.001 (2)
C7	0.044 (3)	0.034 (3)	0.040 (3)	0.003 (2)	0.017 (2)	−0.002 (2)
C8	0.037 (3)	0.040 (3)	0.041 (3)	0.002 (2)	0.010 (2)	0.008 (2)
C9	0.059 (4)	0.051 (3)	0.050 (3)	0.000 (3)	0.021 (3)	0.005 (3)
C10	0.066 (4)	0.055 (4)	0.070 (4)	−0.001 (3)	0.033 (3)	0.023 (3)
C11	0.060 (4)	0.041 (3)	0.081 (4)	−0.006 (3)	0.015 (3)	0.009 (3)
C12	0.099 (5)	0.046 (4)	0.062 (4)	−0.010 (3)	0.032 (4)	−0.004 (3)
C13	0.079 (4)	0.040 (3)	0.052 (3)	−0.009 (3)	0.030 (3)	0.002 (3)
C14	0.048 (3)	0.046 (3)	0.038 (3)	0.003 (3)	0.014 (3)	−0.002 (2)
C15	0.059 (4)	0.033 (3)	0.038 (3)	0.006 (3)	0.012 (2)	−0.006 (2)
C16	0.056 (4)	0.052 (3)	0.053 (3)	0.004 (3)	0.017 (3)	0.002 (3)
C17	0.088 (5)	0.058 (4)	0.056 (4)	0.016 (4)	0.030 (3)	0.016 (3)
C18	0.070 (5)	0.063 (4)	0.053 (4)	0.017 (3)	0.008 (3)	0.004 (3)
C19	0.049 (4)	0.060 (4)	0.079 (4)	0.011 (3)	0.007 (3)	−0.007 (3)
C20	0.058 (4)	0.041 (3)	0.055 (3)	0.006 (3)	0.012 (3)	−0.001 (3)
C21	0.058 (4)	0.046 (3)	0.073 (4)	−0.001 (3)	0.023 (3)	−0.004 (3)
C22	0.058 (4)	0.062 (4)	0.096 (5)	−0.010 (4)	0.022 (4)	−0.008 (4)
C23	0.088 (5)	0.049 (4)	0.081 (4)	−0.020 (4)	0.025 (4)	−0.020 (3)
C24	0.079 (5)	0.037 (3)	0.077 (4)	0.007 (3)	0.015 (4)	−0.008 (3)
C25	0.065 (4)	0.044 (3)	0.058 (3)	0.008 (3)	0.012 (3)	−0.005 (3)
C26	0.052 (3)	0.041 (3)	0.042 (3)	0.002 (3)	0.018 (2)	−0.002 (2)
C27	0.061 (4)	0.046 (3)	0.045 (3)	−0.015 (3)	0.015 (3)	−0.008 (3)
C28	0.063 (4)	0.062 (4)	0.039 (3)	−0.004 (3)	0.014 (3)	0.001 (3)
C29	0.056 (3)	0.050 (3)	0.042 (3)	0.000 (3)	0.019 (3)	0.003 (3)
C30	0.050 (3)	0.044 (3)	0.041 (3)	−0.005 (2)	0.012 (2)	−0.004 (2)
C31	0.046 (3)	0.043 (3)	0.040 (3)	0.004 (2)	0.018 (2)	0.000 (2)
C32	0.044 (3)	0.041 (3)	0.043 (3)	0.003 (2)	0.016 (2)	0.001 (2)
C33	0.051 (3)	0.042 (3)	0.040 (3)	−0.002 (3)	0.005 (2)	0.003 (2)
C34	0.058 (4)	0.057 (4)	0.052 (3)	0.001 (3)	0.009 (3)	−0.008 (3)
C35	0.087 (5)	0.053 (4)	0.064 (4)	0.002 (4)	0.012 (4)	−0.019 (3)
C36	0.080 (5)	0.061 (4)	0.078 (5)	−0.021 (4)	0.001 (4)	−0.014 (4)
C37	0.067 (4)	0.076 (4)	0.072 (4)	−0.023 (4)	0.017 (3)	−0.006 (4)
C38	0.056 (4)	0.052 (3)	0.058 (3)	−0.004 (3)	0.017 (3)	−0.007 (3)
C39	0.048 (3)	0.056 (4)	0.039 (3)	0.011 (3)	0.013 (3)	0.000 (3)
C40	0.051 (3)	0.061 (4)	0.044 (3)	0.010 (3)	0.018 (3)	0.004 (3)
C41	0.071 (4)	0.067 (4)	0.050 (3)	0.010 (3)	0.015 (3)	0.005 (3)
C42	0.086 (5)	0.082 (5)	0.054 (4)	0.015 (4)	0.016 (4)	0.016 (4)
C43	0.082 (5)	0.118 (6)	0.047 (4)	0.010 (5)	0.006 (3)	0.005 (4)
C44	0.091 (5)	0.094 (5)	0.062 (4)	−0.009 (4)	0.019 (4)	0.008 (4)
C45	0.070 (4)	0.070 (4)	0.052 (4)	−0.004 (3)	0.013 (3)	0.006 (3)
C46	0.082 (4)	0.053 (4)	0.047 (3)	0.002 (3)	0.022 (3)	0.002 (3)
C47	0.116 (6)	0.048 (4)	0.050 (4)	−0.003 (4)	0.028 (4)	0.006 (3)
C48	0.095 (5)	0.056 (4)	0.064 (4)	−0.016 (3)	0.037 (4)	−0.001 (3)
C49	0.062 (4)	0.058 (4)	0.059 (4)	−0.006 (3)	0.020 (3)	0.001 (3)
C50	0.059 (4)	0.050 (3)	0.053 (3)	0.002 (3)	0.023 (3)	0.005 (3)

### Geometric parameters (Å, °)

**Table:**

Ni1—O1	1.808 (3)	C19—C20	1.371 (7)
Ni1—O2	1.829 (3)	C19—H19	0.9300
Ni1—N2	1.831 (4)	C20—H20	0.9300
Ni1—N5	1.930 (4)	C21—C22	1.359 (7)
Ni2—O3	1.800 (3)	C21—H21	0.9300
Ni2—N4	1.834 (4)	C22—C23	1.361 (8)
Ni2—O4	1.834 (3)	C22—H22	0.9300
Ni2—N6	1.917 (4)	C23—C24	1.357 (8)
Br1—C4	1.896 (5)	C23—H23	0.9300
Br2—C29	1.899 (5)	C24—C25	1.371 (7)
O1—C1	1.305 (5)	C24—H24	0.9300
O2—C14	1.302 (6)	C25—H25	0.9300
O3—C26	1.310 (5)	C26—C31	1.402 (6)
O4—C39	1.305 (6)	C26—C27	1.408 (7)
N1—C14	1.305 (6)	C27—C28	1.365 (6)
N1—N2	1.404 (5)	C27—H27	0.9300
N2—C7	1.298 (6)	C28—C29	1.390 (7)
N3—C39	1.311 (6)	C28—H28	0.9300
N3—N4	1.405 (5)	C29—C30	1.351 (6)
N4—C32	1.307 (6)	C30—C31	1.413 (6)
N5—C21	1.337 (6)	C30—H30	0.9300
N5—C25	1.338 (6)	C31—C32	1.440 (6)
N6—C50	1.341 (6)	C32—C33	1.508 (7)
N6—C46	1.344 (6)	C33—C38	1.377 (7)
C1—C2	1.395 (6)	C33—C34	1.389 (7)
C1—C6	1.421 (6)	C34—C35	1.379 (8)
C2—C3	1.354 (7)	C34—H34	0.9300
C2—H2	0.9300	C35—C36	1.357 (8)
C3—C4	1.396 (7)	C35—H35	0.9300
C3—H3	0.9300	C36—C37	1.379 (8)
C4—C5	1.359 (6)	C36—H36	0.9300
C5—C6	1.397 (6)	C37—C38	1.356 (7)
C5—H5	0.9300	C37—H37	0.9300
C6—C7	1.455 (6)	C38—H38	0.9300
C7—C8	1.491 (6)	C39—C40	1.465 (7)
C8—C9	1.371 (6)	C40—C41	1.375 (7)
C8—C13	1.372 (6)	C40—C45	1.389 (7)
C9—C10	1.381 (7)	C41—C42	1.383 (8)
C9—H9	0.9300	C41—H41	0.9300
C10—C11	1.366 (7)	C42—C43	1.348 (9)
C10—H10	0.9300	C42—H42	0.9300
C11—C12	1.359 (7)	C43—C44	1.367 (9)
C11—H11	0.9300	C43—H43	0.9300
C12—C13	1.376 (7)	C44—C45	1.388 (8)
C12—H12	0.9300	C44—H44	0.9300
C13—H13	0.9300	C45—H45	0.9300
C14—C15	1.470 (7)	C46—C47	1.374 (7)
C15—C16	1.364 (7)	C46—H46	0.9300
C15—C20	1.374 (7)	C47—C48	1.370 (8)
C16—C17	1.395 (7)	C47—H47	0.9300
C16—H16	0.9300	C48—C49	1.354 (7)
C17—C18	1.364 (8)	C48—H48	0.9300
C17—H17	0.9300	C49—C50	1.362 (7)
C18—C19	1.370 (8)	C49—H49	0.9300
C18—H18	0.9300	C50—H50	0.9300
			
O1—Ni1—O2	177.13 (15)	N5—C21—C22	123.0 (5)
O1—Ni1—N2	95.47 (16)	N5—C21—H21	118.5
O2—Ni1—N2	84.18 (16)	C22—C21—H21	118.5
O1—Ni1—N5	89.70 (16)	C21—C22—C23	119.1 (6)
O2—Ni1—N5	90.88 (16)	C21—C22—H22	120.4
N2—Ni1—N5	173.05 (16)	C23—C22—H22	120.4
O3—Ni2—N4	95.41 (16)	C24—C23—C22	119.1 (6)
O3—Ni2—O4	178.09 (17)	C24—C23—H23	120.4
N4—Ni2—O4	84.71 (16)	C22—C23—H23	120.4
O3—Ni2—N6	89.21 (16)	C23—C24—C25	119.1 (5)
N4—Ni2—N6	175.27 (17)	C23—C24—H24	120.5
O4—Ni2—N6	90.72 (16)	C25—C24—H24	120.5
C1—O1—Ni1	127.2 (3)	N5—C25—C24	122.5 (5)
C14—O2—Ni1	110.5 (3)	N5—C25—H25	118.7
C26—O3—Ni2	127.0 (3)	C24—C25—H25	118.7
C39—O4—Ni2	110.3 (3)	O3—C26—C31	124.8 (4)
C14—N1—N2	107.4 (4)	O3—C26—C27	116.5 (4)
C7—N2—N1	116.0 (4)	C31—C26—C27	118.7 (4)
C7—N2—Ni1	129.5 (3)	C28—C27—C26	122.9 (5)
N1—N2—Ni1	114.5 (3)	C28—C27—H27	118.6
C39—N3—N4	108.2 (4)	C26—C27—H27	118.6
C32—N4—N3	117.0 (4)	C27—C28—C29	117.8 (5)
C32—N4—Ni2	129.1 (3)	C27—C28—H28	121.1
N3—N4—Ni2	113.9 (3)	C29—C28—H28	121.1
C21—N5—C25	117.1 (5)	C30—C29—C28	121.2 (5)
C21—N5—Ni1	120.9 (4)	C30—C29—Br2	119.0 (4)
C25—N5—Ni1	122.0 (4)	C28—C29—Br2	119.8 (4)
C50—N6—C46	116.9 (5)	C29—C30—C31	122.2 (5)
C50—N6—Ni2	121.1 (3)	C29—C30—H30	118.9
C46—N6—Ni2	122.0 (4)	C31—C30—H30	118.9
O1—C1—C2	117.7 (4)	C26—C31—C30	117.3 (4)
O1—C1—C6	124.7 (4)	C26—C31—C32	122.8 (4)
C2—C1—C6	117.7 (5)	C30—C31—C32	119.9 (4)
C3—C2—C1	123.5 (5)	N4—C32—C31	120.8 (4)
C3—C2—H2	118.3	N4—C32—C33	119.5 (4)
C1—C2—H2	118.3	C31—C32—C33	119.6 (4)
C2—C3—C4	118.6 (5)	C38—C33—C34	119.3 (5)
C2—C3—H3	120.7	C38—C33—C32	119.9 (5)
C4—C3—H3	120.7	C34—C33—C32	120.7 (5)
C5—C4—C3	119.8 (5)	C35—C34—C33	120.0 (6)
C5—C4—Br1	120.2 (4)	C35—C34—H34	120.0
C3—C4—Br1	120.0 (4)	C33—C34—H34	120.0
C4—C5—C6	122.6 (5)	C36—C35—C34	119.4 (6)
C4—C5—H5	118.7	C36—C35—H35	120.3
C6—C5—H5	118.7	C34—C35—H35	120.3
C5—C6—C1	117.7 (4)	C35—C36—C37	121.1 (6)
C5—C6—C7	120.3 (4)	C35—C36—H36	119.5
C1—C6—C7	122.0 (4)	C37—C36—H36	119.5
N2—C7—C6	121.1 (4)	C38—C37—C36	119.7 (6)
N2—C7—C8	120.1 (4)	C38—C37—H37	120.2
C6—C7—C8	118.8 (4)	C36—C37—H37	120.2
C9—C8—C13	118.7 (5)	C37—C38—C33	120.6 (5)
C9—C8—C7	120.8 (4)	C37—C38—H38	119.7
C13—C8—C7	120.4 (4)	C33—C38—H38	119.7
C8—C9—C10	120.2 (5)	O4—C39—N3	123.0 (4)
C8—C9—H9	119.9	O4—C39—C40	118.2 (5)
C10—C9—H9	119.9	N3—C39—C40	118.9 (5)
C11—C10—C9	120.4 (5)	C41—C40—C45	118.1 (5)
C11—C10—H10	119.8	C41—C40—C39	120.5 (5)
C9—C10—H10	119.8	C45—C40—C39	121.4 (5)
C12—C11—C10	119.5 (5)	C40—C41—C42	120.4 (6)
C12—C11—H11	120.2	C40—C41—H41	119.8
C10—C11—H11	120.2	C42—C41—H41	119.8
C11—C12—C13	120.3 (5)	C43—C42—C41	120.4 (6)
C11—C12—H12	119.9	C43—C42—H42	119.8
C13—C12—H12	119.9	C41—C42—H42	119.8
C8—C13—C12	120.8 (5)	C42—C43—C44	121.4 (6)
C8—C13—H13	119.6	C42—C43—H43	119.3
C12—C13—H13	119.6	C44—C43—H43	119.3
O2—C14—N1	123.2 (4)	C43—C44—C45	118.3 (7)
O2—C14—C15	117.4 (4)	C43—C44—H44	120.8
N1—C14—C15	119.4 (5)	C45—C44—H44	120.8
C16—C15—C20	119.0 (5)	C44—C45—C40	121.4 (6)
C16—C15—C14	121.6 (5)	C44—C45—H45	119.3
C20—C15—C14	119.3 (5)	C40—C45—H45	119.3
C15—C16—C17	120.4 (5)	N6—C46—C47	122.3 (6)
C15—C16—H16	119.8	N6—C46—H46	118.9
C17—C16—H16	119.8	C47—C46—H46	118.9
C18—C17—C16	119.8 (5)	C48—C47—C46	119.5 (6)
C18—C17—H17	120.1	C48—C47—H47	120.2
C16—C17—H17	120.1	C46—C47—H47	120.2
C17—C18—C19	119.6 (5)	C49—C48—C47	118.4 (6)
C17—C18—H18	120.2	C49—C48—H48	120.8
C19—C18—H18	120.2	C47—C48—H48	120.8
C18—C19—C20	120.4 (6)	C48—C49—C50	119.9 (6)
C18—C19—H19	119.8	C48—C49—H49	120.1
C20—C19—H19	119.8	C50—C49—H49	120.1
C19—C20—C15	120.6 (5)	N6—C50—C49	123.0 (5)
C19—C20—H20	119.7	N6—C50—H50	118.5
C15—C20—H20	119.7	C49—C50—H50	118.5
			
N2—Ni1—O1—C1	1.1 (4)	C15—C16—C17—C18	0.8 (8)
N5—Ni1—O1—C1	176.4 (4)	C16—C17—C18—C19	0.5 (8)
N2—Ni1—O2—C14	3.2 (3)	C17—C18—C19—C20	−1.6 (9)
N5—Ni1—O2—C14	−171.9 (3)	C18—C19—C20—C15	1.5 (8)
N4—Ni2—O3—C26	−1.4 (4)	C16—C15—C20—C19	−0.2 (7)
N6—Ni2—O3—C26	177.5 (4)	C14—C15—C20—C19	−178.6 (5)
N4—Ni2—O4—C39	0.4 (3)	C25—N5—C21—C22	1.5 (8)
N6—Ni2—O4—C39	−178.4 (3)	Ni1—N5—C21—C22	179.4 (4)
C14—N1—N2—C7	−178.1 (4)	N5—C21—C22—C23	0.3 (9)
C14—N1—N2—Ni1	1.6 (5)	C21—C22—C23—C24	−1.1 (9)
O1—Ni1—N2—C7	−0.2 (4)	C22—C23—C24—C25	0.1 (9)
O2—Ni1—N2—C7	176.9 (4)	C21—N5—C25—C24	−2.5 (8)
O1—Ni1—N2—N1	−179.9 (3)	Ni1—N5—C25—C24	179.6 (4)
O2—Ni1—N2—N1	−2.7 (3)	C23—C24—C25—N5	1.8 (9)
C39—N3—N4—C32	−177.2 (4)	Ni2—O3—C26—C31	3.0 (7)
C39—N3—N4—Ni2	1.1 (5)	Ni2—O3—C26—C27	−177.2 (3)
O3—Ni2—N4—C32	−1.0 (4)	O3—C26—C27—C28	178.8 (5)
O4—Ni2—N4—C32	177.1 (4)	C31—C26—C27—C28	−1.3 (8)
O3—Ni2—N4—N3	−178.9 (3)	C26—C27—C28—C29	0.9 (8)
O4—Ni2—N4—N3	−0.8 (3)	C27—C28—C29—C30	0.2 (8)
O1—Ni1—N5—C21	150.0 (4)	C27—C28—C29—Br2	−179.4 (4)
O2—Ni1—N5—C21	−27.2 (4)	C28—C29—C30—C31	−0.8 (8)
O1—Ni1—N5—C25	−32.2 (4)	Br2—C29—C30—C31	178.8 (4)
O2—Ni1—N5—C25	150.7 (4)	O3—C26—C31—C30	−179.5 (4)
O3—Ni2—N6—C50	−29.6 (4)	C27—C26—C31—C30	0.7 (7)
O4—Ni2—N6—C50	152.3 (4)	O3—C26—C31—C32	−2.0 (8)
O3—Ni2—N6—C46	152.2 (4)	C27—C26—C31—C32	178.1 (5)
O4—Ni2—N6—C46	−25.9 (4)	C29—C30—C31—C26	0.3 (7)
Ni1—O1—C1—C2	177.6 (3)	C29—C30—C31—C32	−177.2 (5)
Ni1—O1—C1—C6	−2.5 (7)	N3—N4—C32—C31	179.8 (4)
O1—C1—C2—C3	176.3 (4)	Ni2—N4—C32—C31	1.9 (7)
C6—C1—C2—C3	−3.6 (7)	N3—N4—C32—C33	−0.8 (6)
C1—C2—C3—C4	0.9 (8)	Ni2—N4—C32—C33	−178.6 (3)
C2—C3—C4—C5	1.5 (7)	C26—C31—C32—N4	−0.4 (7)
C2—C3—C4—Br1	−177.8 (4)	C30—C31—C32—N4	176.9 (4)
C3—C4—C5—C6	−1.1 (7)	C26—C31—C32—C33	−179.9 (4)
Br1—C4—C5—C6	178.2 (3)	C30—C31—C32—C33	−2.6 (7)
C4—C5—C6—C1	−1.6 (7)	N4—C32—C33—C38	118.7 (5)
C4—C5—C6—C7	179.4 (4)	C31—C32—C33—C38	−61.9 (6)
O1—C1—C6—C5	−176.1 (4)	N4—C32—C33—C34	−64.9 (6)
C2—C1—C6—C5	3.8 (6)	C31—C32—C33—C34	114.6 (5)
O1—C1—C6—C7	2.9 (7)	C38—C33—C34—C35	0.2 (8)
C2—C1—C6—C7	−177.2 (4)	C32—C33—C34—C35	−176.3 (5)
N1—N2—C7—C6	−179.7 (4)	C33—C34—C35—C36	−0.3 (9)
Ni1—N2—C7—C6	0.7 (7)	C34—C35—C36—C37	0.2 (10)
N1—N2—C7—C8	0.5 (6)	C35—C36—C37—C38	0.0 (10)
Ni1—N2—C7—C8	−179.2 (3)	C36—C37—C38—C33	0.0 (9)
C5—C6—C7—N2	177.0 (4)	C34—C33—C38—C37	−0.1 (8)
C1—C6—C7—N2	−1.9 (7)	C32—C33—C38—C37	176.4 (5)
C5—C6—C7—C8	−3.1 (6)	Ni2—O4—C39—N3	0.1 (6)
C1—C6—C7—C8	178.0 (4)	Ni2—O4—C39—C40	−178.9 (3)
N2—C7—C8—C9	−74.7 (6)	N4—N3—C39—O4	−0.8 (6)
C6—C7—C8—C9	105.4 (5)	N4—N3—C39—C40	178.2 (4)
N2—C7—C8—C13	107.3 (5)	O4—C39—C40—C41	12.4 (7)
C6—C7—C8—C13	−72.5 (6)	N3—C39—C40—C41	−166.6 (5)
C13—C8—C9—C10	1.9 (7)	O4—C39—C40—C45	−169.5 (5)
C7—C8—C9—C10	−176.1 (5)	N3—C39—C40—C45	11.5 (8)
C8—C9—C10—C11	−1.0 (8)	C45—C40—C41—C42	−0.8 (8)
C9—C10—C11—C12	−0.4 (9)	C39—C40—C41—C42	177.3 (5)
C10—C11—C12—C13	0.9 (9)	C40—C41—C42—C43	0.6 (10)
C9—C8—C13—C12	−1.3 (8)	C41—C42—C43—C44	0.5 (11)
C7—C8—C13—C12	176.6 (5)	C42—C43—C44—C45	−1.2 (10)
C11—C12—C13—C8	−0.1 (9)	C43—C44—C45—C40	0.9 (10)
Ni1—O2—C14—N1	−3.5 (6)	C41—C40—C45—C44	0.1 (9)
Ni1—O2—C14—C15	175.5 (3)	C39—C40—C45—C44	−178.0 (5)
N2—N1—C14—O2	1.3 (6)	C50—N6—C46—C47	−0.9 (7)
N2—N1—C14—C15	−177.7 (4)	Ni2—N6—C46—C47	177.4 (4)
O2—C14—C15—C16	−141.4 (5)	N6—C46—C47—C48	−0.8 (9)
N1—C14—C15—C16	37.6 (7)	C46—C47—C48—C49	1.0 (9)
O2—C14—C15—C20	36.9 (6)	C47—C48—C49—C50	0.3 (9)
N1—C14—C15—C20	−144.1 (5)	C46—N6—C50—C49	2.3 (7)
C20—C15—C16—C17	−1.0 (7)	Ni2—N6—C50—C49	−176.0 (4)
C14—C15—C16—C17	177.4 (5)	C48—C49—C50—N6	−2.1 (8)
